# Hospital Preparedness Challenges in Biological Disasters: A Qualitative Study

**DOI:** 10.1017/dmp.2020.434

**Published:** 2020-11-05

**Authors:** Mohsen Aminizadeh, Mehrdad Farrokhi, Abbas Ebadi, Gholamreza Masoumi, Pirhossein Kolivand, Hamidreza Khankeh

**Affiliations:** 1 Health in Emergency and Disaster Research Center, University of Social Welfare and Rehabilitation Sciences, Tehran, Iran; 2 Health in Emergency and Disaster Research Center, Kerman University of Medical Sciences, Kerman, Iran; 3 Behavioral Sciences Research Center, Life Style Institute, Faculty of Nursing, Baqiyatallah University of Medical Sciences, Tehran, Iran; 4 Trauma and Injury Research Center, Iran University of Medical Sciences, Tehran, Iran; 5 Ministry of Health and Medical Education, Tehran, Iran; 6 Department of Clinical Science and Education, Karolinska Institute, Stockholm, Sweden

**Keywords:** hospital preparedness, biological disaster, biological events, COVID-19, content analysis

## Abstract

**Objective:**

Identification of hospital preparedness challenges against biological events such as coronavirus disease 2019 (COVID-19) is essential to improve dynamics, quality, and business continuity confidence in the health system. Accordingly, the purpose of the present study is to evaluate the challenges of hospital preparedness in biological events.

**Methods:**

This study used a qualitative method using content analysis in which 20 health-care managers and experts who are experienced in biological events were selected through purposeful sampling. The data collection was done through semi-structured interviews, which continued until data saturation. The data were analyzed using qualitative content analysis as well as the Landman and Graneheim Approach.

**Results:**

Six main concepts (training and practice, resource management, safety and health, patient management, risk communication, and laboratory and surveillance) and 14 subconcepts were extracted on hospital preparedness challenges in biological events through analyzing interviews.

**Conclusions:**

The present study indicated that the health system of the country faces many challenges in response to biological events and threats. Moreover, study participants indicated that Iranian hospitals were not prepared for biological events. It is recommended to design preparedness plans of hospitals based on preparedness standards for biological events. In addition, comprehensive measures are required to enhance their capacity to respond to biological emergencies.

Incidents caused by biological events are a serious threat to the health and safety of citizens, which can impose a huge financial and social burden on the affected community and health systems. The potential consequences and costs of not being prepared for such events can be overwhelming.^1^ Biological disasters are a serious threat to the health and safety of citizens, which can impose significant financial and labor burdens on the affected community and health systems, and the potential consequences and costs of not being prepared for such events can be staggering.^2^ In November 2019, a novel coronavirus disease 2019 (COVID-19) was first reported and then became widespread within Wuhan, the capital city of Hubei Province of China.^3^ The World Health Organization (WHO) named the COVID-19 outbreak associated with transmission of the novel coronavirus severe acute respiratory syndrome coronavirus 2 (SARS-CoV-2) a public health emergency of international concern.^4^ The pandemic poses a potent economic threat as well as a public health crisis. The virus has rapidly spread across continents, with more than 2,501,919 confirmed cases and more than 171,741 deaths by April 21, 2020.^5^ As of 12 March 2020, According to the report of Iran’s health ministry, there had been 5,297 COVID-19 deaths in Iran with a total of around 84,802 confirmed infections of which 60,965 have recovered.^6^

Therefore, the rapid and appropriate response to such incidents (anthrax, Ebola, COVID-19, etc.) can play an important role in reducing the harmful effects of these events on physical health and greatly reduce its psychological effects.^2^ The WHO has identified preparedness as an important part of the sustainable development process and emphasized the implementation of required activities.^7^ Despite these widespread efforts and advances in hospital preparedness to manage biological events, researchers have declared that a large proportion of hospitals are not yet well prepared for such incidents and hospitals face numerous challenges given the different pathophysiology of these events.^8,9^ The health system of every country is responsible for protecting the safety and health of human as the first and foremost demand. Iran, as a member state of WHO, has taken positive steps to prevent, protect, control, and prepare public health responses to the international spread of disease in line with the objectives of the 2005 International Health Regulations.^10^

Response to diseases caused by biological events is still 1 of the major health issues in developing countries that need to be addressed. Iran is located in the eastern Mediterranean region of the WHO^2^ with neighbors that do not yet have a dynamic, active, and coherent health system. Given the emergence of biological events in Iran, such as the outbreak of influenza, the Crimean Congo fever, and the 2019-2020 coronavirus pandemic, the need for preparing hospitals for biological events in response to these incidents at local and national levels is felt. This study aims to identify the challenges of hospital preparedness in biological events in Iran by a qualitative study. The information from this study can provide a perspective for health-care policy-makers and managers in future planning to address the identified challenges of health-care centers and effective regional, national response to biological events.

## Methods

The purpose of qualitative study is to identify the challenges of hospital preparedness in biological events from the perspective of research participants with purposive sampling to achieve maximum variation (in terms of age, gender, education, operational experience, and organizational class) through a semi-structure interview of experts in the field of emergency and disaster with awareness, knowledge, and experience in infectious disease and biological events such as influenza and coronavirus outbreaks and hospital preparedness. Interviews lasted 40-70 min (50 min on average). A guide for open question interview was used in accordance with subcategories predetermined. Furthermore, the observation of interviewer was recorded as field notes.

The selection process of samples and collecting data continued until the saturation of the data was reached. Lundman and Graneheim 5-step analysis method was used to analyze the content of qualitative data. The 4 criteria of credibility, dependability, confirmability, and transferability were used to ensure data validity and reliability.^11^ To increase credibility, the constant comparison, active listening, prolonged engagement with data, immersion in data, as well as data source and investigator triangulation techniques were used. To ensure the dependability of the findings, we documented and kept a record of our analytic activities for the purpose of audit trailing. The confirmability of the study findings was ensured by using peer-checking and member-checking techniques. We also strived to recruit a sample with maximum variation to improve the transferability of the study findings. Ethical approval of this study was obtained from the Ethics Committee of University of Social Welfare and Rehabilitation Sciences in Tehran, Iran (IR.USWR.REC.1398.030). To manage data, MAXQDA (version 10) software was used. To fully understand the contents, interview transcripts were read several times to produce an overall impression of the data and the most important meaning units were determined at the same time. Then coding was done and the codes put in the right place in predetermined categories

## Results

A total of 20 emergency management experts, including risk committee secretaries, policy-makers, faculty members and staff with scientific and practical experience in biological events and threats, were interviewed (Table 1).

**Table 1.** Characteristics of managers, policy-makers, faculty members, and staff participating in the study

Participant number	Gender	Age	Education	Service location
Participant 1	Female	45	BS of Nursing	Afzalipour Hospital in Kerman
Participant 2	Female	39	Master of Nursing	Afzalipour Hospital in Kerman
Participant 3	Male	46	BS of Nursing	Masih Daneshvari Hospital in Tehran
Participant 4	Male	42	Infectious Diseases Specialist	Afzalipour Hospital in Kerman
Participant 5	Male	44	Infectious Diseases Specialist	Ministry of Health
Participant 6	Male	52	PhD in Nursing	Ministry of Health
Participant 7	Male	40	Infectious Diseases Specialist	Imam Khomeini Hospital in Tehran
Participant 8	Male	43	Emergency Medicine Specialist	Emergency Organization
Participant 9	Female	47	PhD in Laboratory Sciences	Ministry of Health
Participant 10	Male	39	PhD in Human Management	Hospital management of Afzali Hospital in Kerman
Participant 11	Male	37	Infectious Diseases Specialist	Masih Daneshvari Hospital in Tehran
Participant 12	Male	50	General Practitioner	Afzali Hospital in Tehran
Participant 13	Male	49	General Practitioner	Kerman University of Medical Sciences
Participant 14	Female	36	BS of Nursing	Afzalipour Hospital in Kerman
Participant 15	Male	38	PhD of Health in Disasters	Tehran Emergency
Participant 16	Male	44	PhD of Health in Disasters	Kerman University of Medical Sciences
Participant 17	Male	35	Emergency Medical Expert	Tehran Emergency
Participant 18	Male	35	Master of Special Nursing	Iran University of Medical Sciences
Participant 19	Female	49	Senior Defense Expert	Baqiyatallah University of Medical Sciences
Participant 20	Female	36	Environmental Health Expert	Kerman University of Medical Sciences

Six main concepts and 14 subconcepts were extracted in the field of hospital challenges in biological events from the analysis of the interviews. The extracted challenges included education and training, resource management, patient management, risk communication, safety and health, laboratory, and surveillance (Table 2).

**Table 2.** Main concepts and subconcepts of hospital preparedness challenges in biological events

Main Categories	Subcategories	
Education and training	Inefficient training	
	Scenic practices	
Resource management	Staff management	Motivational factors
		Organizational factors
		** **Individual factors
	Surge capacity (equipment and structure)	
Patient management	Biological triage	
	Treatment management	
Risk communication	Perception of risk	
	Informing	
Safety and health	Environmental safety	
	Individual safety	
Laboratory and surveillance	Syndromic surveillance system	
	Laboratory detection capability	

### Education and Training

Ineffective training and inefficient practices were among the identified challenges in this regard. According to the experiences of the interviewees, training was considered as one of the most important factors in preparing hospitals for biological events, but they state that courses of training are not effective enough and most of the staff ignore it. Participants also complained about the lack of specialist experts for training in biological events. They said that courses of training were conducted to earn points and set the task. One of the participants stated, (P.3)

‘‘*The training has not been profound and forward-looking, and most of the training in this area is sometimes superficial and does not have the necessary effectiveness. Unfortunately, what we are seeing is high-cost training, most of which is scenic and promotional with no output for our hospital. We need to use experts in the field to monitor and evaluate maneuvers.*”

### Resource Management

Interviewees raised 2 subcategories of staff management (motivational factors and individual factors) and surge capacity (equipment and structure) related to resource management. Some participants commented on the motivational factors of staff that there is not proportionality between the staff evaluation and their encouragement process, and they are often discriminated against.

Interviewees identified factors, such as fear of illness, the stress of patient care, infection, and disease malingering, reduction of medical staff participation in outbreaks and epidemics, and decreased social participation, as the individual factors related to staff management.

Some participants confirmed that the architecture and structure of the hospital were not designed for the hospital and that proper ventilation in the hospital space, which is one of the most important measures in biological events, was not done properly. One of the main challenges for hospitals in biological disasters was increasing capacity. Participants noted the cancellation of unnecessary operations and the multifunctional use of space and hospital wards to increase capacity in this area. The creative use of hospital spaces and the establishment of convalescent homes were also mentioned, and experiences of such measures were presented. One of the participants stated that, (P.5)

“*We are in a difficult position in terms of infrastructure; not only did we not have the space to accept these patients. We also face challenges in terms of respiratory isolation, proper ventilation and personal protective equipment, and decontamination of patients.*”

One of the points made by some participants was the creative and innovative production of personal protective equipment such as masks and hospital clothing and the lack of personal protective equipment, and the use of nanotechnology to produce equipment by knowledge-based companies. The shortage of equipment and structure in this research can be the deficiency of isolation and negative pressure rooms, shortage of laboratory capacity of hospital, the shortage of personal protective equipment, shortage of decontamination infrastructure, inadequate positioning, and access to hospitals.

### Patient Management

Patient management was another identified challenge in this research. According to the research participants, 2 subconcepts of biological triage and treatment management were identifiable in this regard. Interviewees indicated that most hospitals did not have the proper infrastructure to triage patients faced with biological events, and screening for patients is not done properly. Biological triage has a different mechanism than other triage methods.

Some participants also stated that the treatment management of patients in biological events, such as emerging and re-emerging diseases, is a challenge due to the different and unknown nature and pathology of these diseases and inadequate knowledge of personnel in managing these events. According to the participants, one of the important challenges in this area is improper and unreasonable prescription for these patients, which leads to microbial resistance. Interviewees believed that antibiotics are widely and nonstandardly prescribed for patients in hospitals. In this regard, there is no regulatory system for prescribing antibiotics.

### Safety and Health

Another extracted challenge was safety and health for which 2 subcategories of environmental safety and personal safety were identified. What the interviewees said in relation to environmental safety indicates that there is no clear audit of the occupational health and safety management system to comply with the criteria, standards, and rules. In this regard, one of the participants stated on occupational safety and health,

"When the workforce is concerned about occupational safety and health and constantly unhappy with the situation, how can you expect things to go well? It manifests itself more in biological events like infectious, a number of our colleagues died or became ill due to lack of personal equipment, this is really worrying.” (Participant No. 12).

Other cases, such as common access for suspected or affected patients and other referring people to the hospital (visitors), unfamiliarity with personal protective equipment, poor quality personal protective equipment, and unfamiliarity with safety principles, were some of the issues mentioned by the individual safety participants.

### Risk Communication

Other challenges identified in these events were risk communication of which 2 subcategories of risk perception and informing were extracted. Participants stated that informing and proper communication is among the effective factors in reducing fear and anxiety of the community and preventing the spread of rumor in managing biological events. One of the challenges they stated was that managers and staff are not familiar with biological events and do not take them seriously. They also do not have a proper understanding of biological events and threats and do not properly understand the significance of these events. Unfamiliarity of staff and community with biological events; ineffective communication with patients, staff, and community; fatalistic view of managers and authorities on incidents; and multi-risk approach of managers and policy-makers were items related to the perception of risk.

Another subcategory of risk communication was informing. One of the main challenges from the point of view of participants was the lack of proper informing to the community due to security concerns and a low amount of reporting. Participants insisted that biological events should get out of security concerns and be treated like other events with transparent informing to increase the perception of risk, managing the rumors, and managing the patients who rush to the hospital.

### Laboratory and Surveillance System

Another challenge was the laboratory and the surveillance system of which 2 subcategories of the syndromic surveillance systems and laboratory detection capability were obtained from the interviewees’ opinions. Participants mentioned factors, such as lack of development of the syndromic surveillance system, lack of integration of the syndromic surveillance system, weakness in the detection capability, timely detection of outbreaks, lack of feedback system, and inaccurate medical recording, as the challenges of the surveillance system. They believed that the surveillance system failed to identify and detect emerging outbreaks and diseases, despite its enormous capacity, and did not achieve much success in this area. Interviewees stated that the syndromic surveillance system software is a very suitable tool for recording diseases caused by biological events, but it does not have the necessary integration while being implemented throughout the country. In this regard, 1 of the participants stated on surveillance system,

“Syndromic surveillance system has been defined in hospitals, but it’s not in the triage department, available to the infection control nurses. Therefore, the patient will not be detected in the triage department and if detected, the identification and data recording will be delayed.” (Participant No. 15).

According to the participants, the limitation of the detection capacity of the reference laboratories, inadequate laboratory capacity at the local level, the lack of linkage between the laboratories, and the lack of an integrated laboratory information system were challenges of the laboratory.

## Discussion

The results show that most interviewees realized the importance of training and practical programs for health-care providers, and they stated that different types of training programs are provided in hospitals, but these courses of trainings and practical programs do not have the necessary impact on hospital preparedness. Numerous studies have indicated that training programs do not have the appropriate capability and effectiveness.^8,12,13^ Given the nature of biological events that require specialized expertise, training should be provided by professional trainers with the help of up-to-date methods and simulators. Simulation practices are more effective to enhance the preparedness of the organization and staff in response to disasters because people are in the same position.

Surge capacity (equipment and structure) was another challenge in the field of hospital preparedness for biological events. Some of the actions that can help in this regard are the discharge of elective and outpatient patients, the cancellation of nonemergency surgeries and the call for troops, determining and identifying alternative patient care, space prediction for cohort isolation, predictions and measures to increase capacity in special care units (eg, ICU). Also, the use of auxiliary places, such as stadiums and schools, in the present study has been proposed as surge capacity strategies that can be very effective and helpful in the process of management and treatment of respiratory patients in these accidents.

Facilities, equipment, human resources, and ancillary spaces should be considered as capacity building in advance. The WHO 2020 stated, due to the COVID-19 pandemic, protective equipment is a major challenge in hospitals.^4^

Environmental and individual safety is the identified challenges related to safety and health in this study. Studies have indicated that nurses’ willingness to respond to dangerous incidents, such as biological events, can depend on their personal and family safety in addition to their clinical competencies, which should be considered.^14^ During the outbreak periods of COVID-19 or other infectious diseases, the implementations of infection prevention and control (IPC) becomes a great importance in health-care settings.^9^ Recently, Wu et al. (2020),^15^ have reported the problems of COVID-19 IPC in health-care settings, particularly highlighting the problems of personal protection of health-care workers. In this regard, applying IPC standards in hospitals can reduce the potential risks to patients, staff, and referring people, which will ultimately increase the efficiency, effectiveness, and performance of hospitals.^2^

One of the newly identified issues in this research was biological triage. The main purpose of this triage is to reduce the transmission of infection of people with high-priority treatment, emphasizing the highest amount of services in the shortest possible time to patients caused by biological accidents. Studies have also shown that microbes become highly resistant to overconsumption of antibiotics. The study by Hormozi et al. (2018) showed that antibiotic resistance has an increasing trend, and strategic measures of prevention are needed to reduce nosocomial infections.^16^ Therefore, a positive step can be taken to improve hospital preparedness by IPC programs, such as hand hygiene, sterilization, cleaning, disinfection, personal protective equipment, and restrictions on the wide use of antibiotics and developing treatment protocols for antibiotic use, as well as developing biological triage and appropriate treatment protocols.

According to the interviewees, hospitals do not have the capacity to detect outbreaks at an early stage despite the syndromic surveillance system. Li et al., (2008) stated that early detection and identification of diseases caused by biological events such as public health emergencies is one of the important goals of health centers for rapid and effective response, which is also a prerequisite for selecting appropriate measures for prevention and treatment in such cases.^17^

One of the most important activities to improve the capability to fight outbreaks is to integrate the syndromic surveillance system with the laboratory information system, the feedback system, develop the diagnostic capacity of the reference and local laboratories, and further monitoring.

## Conclusions

Interviewees’ experience indicated that Iran’s hospitals, like many hospitals around the world, are not prepared for biological events such as COVID-19. The preparedness plan should be designed based on the capacity and leveling of hospitals, identical national protocols, and the standards for preparedness in biological disasters. On the other hand, providing various strategies, including effective training; proper management of resources; proper safety system deployment; enhancing risk perception for managers, staff, and referring people; timely informing; development of syndromic surveillance system; increasing the laboratory detection capacity; and proper patient management, can provide the necessary background for hospital preparedness in these events. Generally, all hospitals in Iran must increase their preparedness for biological disasters, and comprehensive measures are required to enhance their capacity for biological emergencies. In addition, the results of the current study could be used as a basis for designing and developing a standard assessment tool for hospital preparedness in biological events.

### Limitations

According to the characteristics of the Characteristics of Qualitative Research, the generalization of the results of this study is limited only to the study environment. Therefore, a similar study was conducted in other departments, such as hospitals and the emergency department, are recommended.
